# Development of a Hospital Compounded, Taste-Masked, Temozolomide Oral Suspension and 5-Year Real-Life Experience in Treating Paediatric Patients

**DOI:** 10.3390/ph15050555

**Published:** 2022-04-29

**Authors:** Maxime Annereau, Mélanie Hinterlang, Hugues Bienayme, Gilles Vassal, Antoine Pinon, Mathieu Schmitt, Lucas Denis, Caroline Lemarchand, Laurent Martin, François Lemare, Samuel Abbou, Jérémy Bastid, Lionel Tortolano

**Affiliations:** 1 Clinical Pharmacy Department, Gustave Roussy Cancer Campus, 114 rue Edouard Vaillant, 94800 Villejuif, France; melanie.hinterlang@gmail.com (M.H.); antoine.pinon@cea.fr (A.P.); lucas.denis@gustaveroussy.fr (L.D.); francois.lemare@gmail.com (F.L.); 2 EA 401: Matériaux et Santé, Université Paris-Sud, UFR Pharmacie, 92290 Châtenay-Malabry, France; lionel.tortolano@aphp.fr; 3 Orphelia Pharma, 85 Boulevard Saint-Michel, 75005 Paris, France; hugues.bienayme@orphelia-pharma.eu (H.B.); mathschmitt@gmail.com (M.S.); caroline.lemarchand@orphelia-pharma.eu (C.L.); laurent.martin@orphelia-pharma.eu (L.M.); jeremy.bastid@orphelia-pharma.eu (J.B.); 4 Oncopaediatric Department, Gustave Roussy Cancer Campus, 114 rue Edouard Vaillant, 94800 Villejuif, France; gilles.vassal@gustaveroussy.fr (G.V.); samuel.abbou@gustaveroussy.fr (S.A.); 5 Département de Pharmacie, Hôpital Universitaire Henri Mondor, AP-HP, F-94010 Créteil, France

**Keywords:** remozolomide, antineoplastic drugs, pediatric drug, RP-HPLC, stability study, pediatric oncology, oral suspension

## Abstract

The development of oral pediatric forms by pharmaceutical companies is still insufficient. In fact, many drugs used in paediatric oncology, such as temozolomide, are not labeled and adapted for paediatric use. Temozolomide (TMZ) is an alkylating agent used as the standard of care for many adult and pediatric brain tumours, such as neuroblastoma, glioblastoma and medulloblastoma. The present study was carried out to propose a suitable and palatable formulation of the oral liquid preparation of TMZ. The suspension is composed of TMZ suspended in SyrSpend SF pH 4, as well as TMZ crystallization stabilizing agents and sweetening agents. To reach this formulation, several taste-masking agents were evaluated. Here, we describe the method of preparation of the formation as well as the monocentric population treated with the formulation over a 5–year period. A 20 mg/mL TMZ suspension was developed. TMZ suspension is stable for 6 weeks, stored between 2 and 8 degrees, protected from light, and compatible with nasogastric tubes. Thirty-eight patients participated in the palatability study and choose cola flavour, and 104 patients were treated in Gustave Roussy with the developed suspension; no unexpected event was reported. To conclude, we propose here a new TMZ liquid formulation which is stable for at least 6 weeks and well-tolerated with extensive feedback.

## 1. Introduction

Children usually have difficulties in taking medicines in the form of tablets or capsules. The European Medicine Agency (EMA) has recognized that solid oral forms such as hard-gel capsules are not adapted to the pediatric population and are contraindicated in very young children [1]. This problem is even more critical when patients are very young (e.g., patients with neuroblastoma) or their ability to swallow is reduced (brain tumours).

Nowadays, despite international recommendations for developing pediatric oral drug formulations, some drugs still do not exist in liquid oral forms or powder to be dissolved in water prior to administration. In some cases, the poor chemical stability of the drug substance in a solution can explain the lack of development of such forms. Beyond the difficulties of administration, inappropriate formulations limit the potential of dose adaptation in the paediatric population for drugs with a narrow therapeutic index.

Temozolomide (TMZ) is an alkylating agent approved for first-line treatment of adult glioblastoma multiform (GBM) and for second-line treatment of adult and childhood malignant glioma (above the age of three) such as GBM and anaplastic astrocytoma in combination with radiotherapy. TMZ is also widely used in combination with topotecan (TOTEM [2] or with irinotecan (TEMIRI [3]) to treat relapsed or refractory neuroblastoma and medulloblastoma. Furthermore, several ongoing clinical trials are evaluating TMZ in combination with irinotecan for upfront treatment of high-risk Ewing sarcoma (NCT01864109). TMZ is also increasingly used as maintenance therapy or in the context of palliative care.

Candidates for temozolomide treatment are often very young, below 6 years old for neuroblastoma [4,5], and TMZ capsules are too large for their swallowing ability. For these young patients unable to swallow capsules, clinicians have two options: deliver TMZ via intra-venous perfusion, which is much more expensive than the oral route [6] and affects the child’s quality of life by requiring prolonged hospitalization, or ask the parents to open the TMZ capsules and mix the powder content in a fruit compote, a yoghurt or a drink. The latter option is the most widely used. However, it is the least safe mode of administration as it exposes the caregivers and the family to a hazardous drug. In addition, the effective dose administered depends on the mixedure and its complete consumption, which may be compromised given the bitter and metallic taste of TMZ. Finally, as TMZ is unstable under light and at alkaline pH 7, mixing the capsule’s contents with food may result in drug degradation leading to underexposure, which is clearly not acceptable for anticancer therapy.

It is therefore essential to have an oral liquid formulation that is palatable, can be safely administered, and is compatible with a nasogastric tube use. The EMA Draft Inventory of paediatric therapeutic needs [7] (EMA/381728/2014) highlighted the need for an age-appropriate formulation of TMZ which would allow dose flexibility for the paediatric population and guarantee stability, homogeneity, and a low volume of administration. Here, we describe the development of an oral compounded suspension of TMZ prepared by the Gustave Roussy pharmacy from TMZ capsules. For easy daily use, this preparation was developed with the best compromise between chemical stability, dosing accuracy, and volume minimization. Because TMZ is known to have a strong metallic taste, the development required an adequate taste-masking strategy. A palatability and acceptability study was thus performed in order to select the best flavouring agent. Paediatric and adult patients have been treated with the hospital compounded TMZ suspension prepared at Gustave Roussy cancer centre since 2015 and we report here the data reflecting 5 years of its use.

## 2. Results

### 2.1. Preparation of TMZ Suspension

The TMZ oral suspension was prepared in conformity with the guidelines available for hospital-compounded preparations [8] from the TMZ capsule content (see Materials and Methods).

A compatibility study of TMZ and the dry excipients using differential scanning calorimetry (DSC) analysis shows no incompatibility between TMZ and the new dry excipients used in the reformulation. There was no difference between the DSC curves of TMZ alone or combined with the excipients (Figure S1, Supplementary Materials).

TMZ suspension was prepared with commercial TMZ caps, mixed with the excipients presented in Table 1. Citric acid is required for molecule stability at low pH. A taste masking agent and a sweetening agent were necessary to obtain a taste accepted by patients. The final TMZ concentration was 20 mg/mL (Table 1). Attempts to achieve higher TMZ concentrations were not successful. Given the known instability of TMZ under light and high temperatures [9], the suspension was stored in amber glass bottles and stored between 2 and 8 °C.

The general appearance of the TMZ suspension was monitored over the stability-testing period (60 days). The TMZ suspension was white to pink, slightly bright and fluid. No phase shifting or precipitations were observed in any of the samples tested over the 60 days.

### 2.2. Chemical Stability

The chemical stability was evaluated using reversed-phase high-performance liquid chromatography (RP-HPLC), which provides a good separation coefficient (Rs = 12,6) between TMZ and its major degradation product, 5-Aminoimidazole-4-carboxamide (AIC), and allows their quantification in a SyrSpend^®^ matrix (Table 2).

Before evaluating the drug product stability, several forced degradation conditions were assessed in order to identify all resulting degradation products following ICH Q1 A [10]: hydrolytic, oxidative, photolytic and thermal stress conditions. Whatever the forced degradation test used, AIC was the only degradation product. TMZ was particularly stable in acidic hydrolytic and oxidative conditions with 98.5% +/−1.2% and 99.8% +/−1.1% of the initial product after 30 min, respectively.

**Figure 1:**
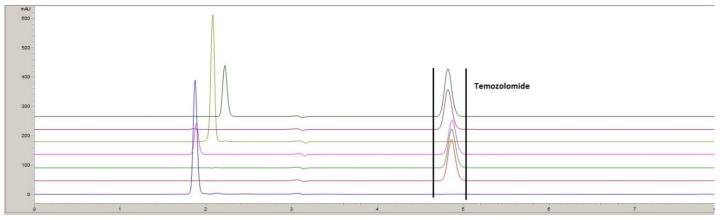
TMZ forced-degradation products chromatograms. Typical chromatogram of AIC reference (blue), TMZ reference (dark red), TMZ under acidic hydrolytic, HCL 1 N (green), TMZ under light exposure (i.e., 2 h of sun test) (pink), TMZ under basic conditions NaoH 1 N (in light green), TMZ under thermal stress 60 °C, 24 h (purple), TMZ under oxidative conditions H_2_O_2_, 1% (dark green).

As expected, TMZ is unstable in basic conditions (NaOH 1 N) at room temperature after 30 min and is totally transformed into AIC. Light (sun test) or high temperature (60 °C) exposure also resulted in degradation into AIC (Figure 1).

**Figure 2:**
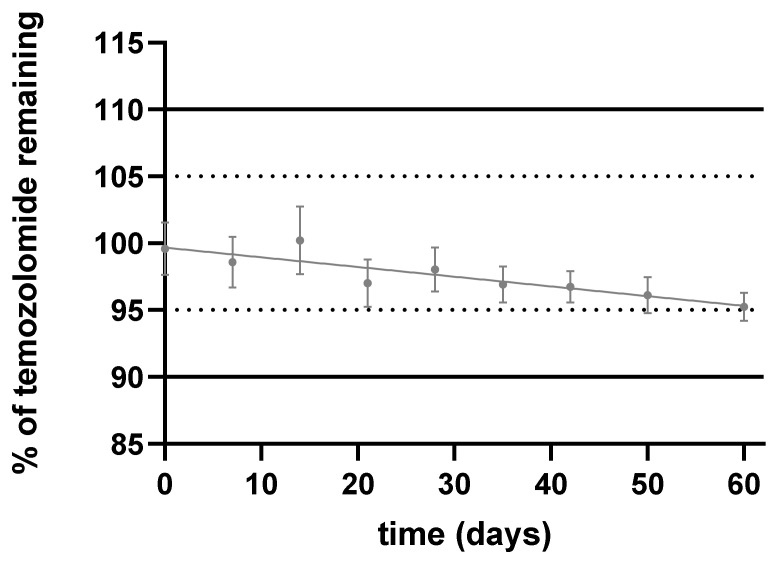
Chemical stability of TMZ suspension stored at + 2–8 °C and protected from the light.

The chemical stability of the TMZ suspension was then evaluated. The acceptance limits for TMZ suspension stability were defined as a concentration comprised within 100 ± 5% and less than 1% of AIC. At + 2–8 °C in amber glass bottles, TMZ content decreased progressively over days but remained within prespecified acceptance limits until D60 (Figure 2). The pH remained stable over the 60-day period. The AIC level was under 1% of the TMZ level (0.68–0.89%).

### 2.3. Microbiological Stability

We first demonstrated that TMZ did not inhibit bacterial (Staphylococcus aureus, Bacillus subtilis, Pseudomonas aeruginosa) and fungal (Candida albicans) growth with the references from the 10th Edition of the European Pharmacopeia [11] (Ph. Eur. 2.6.12). Microbiological quality was evaluated in TMZ suspensions stored at 2–8 °C at days D0, D7, D14, D28 and D60 and showed no bacteriological or fungal contamination.

### 2.4. Compatibility and Stability in Syringes for Oral Use

The forced extraction tests allow the analysis of additives and degradation products of polymers. Our results showed that only an antioxidant and no degradation products were present in the syringes. The chromatographic characteristics of this additive were those of irgafos 168, a well-known antioxidant commonly used to improve the stability of medical devices made of olefin polymers.

We obtained the same extractible profiles for all syringes before or after 10 days of storage with our contact with the oral suspension of temozolomide. Any degradation products of irgafos 168 appeared after storage, thus indicating that there is no interaction.

TMZ suspensions remained chemically stable over 10 days in polypropylene oral use syringes at +2–8 °C. After 10 days in those conditions, 98.6% of the initial concentration of TMZ remained and AIC was under LOD. Microbial quality was also tested in the syringe after 10 days and no bacteriological or fungal contamination was observed at room temperature and at +2–8 °C. Furthermore, our results did not show any traces of polymer, irgafos 168 or its degradation products in the temozolomide suspension.

Altogether, according to those data, TMZ suspension in oral syringes kept under refrigerated conditions and protected from light, is stable for 10 days.

### 2.5. Administration through Nasogastric Tubes

Adsorption of the TMZ suspension on a nasogastric tube was evaluated by running 200 mg TMZ suspension (10 mL of suspension) through a nasogastric tube. After one or two rinsing cycles of the nasogastric tube with 5 or 10 mL of water, 100.0% of the dose was systematically delivered in all testing conditions (<0.05% of residual TMZ extracted from the nasogastric tubes) and AIC was not detectable. The results confirmed that TMZ suspension can be administered through nasogastric tubes (Table 3).

### 2.6. TMZ Administration

**Figure 3:**
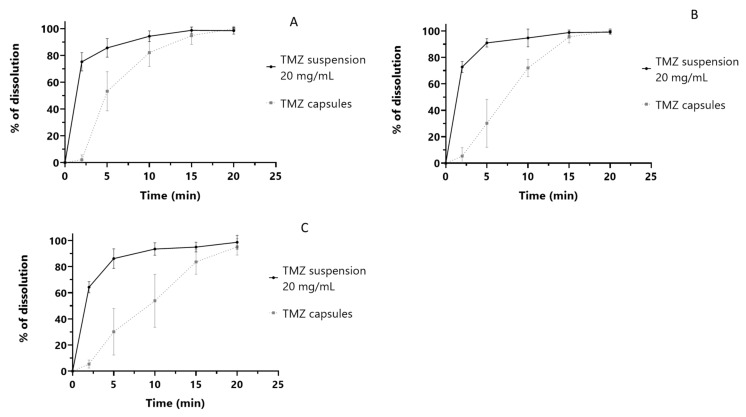
In vitro dissolution profiles of TMZ suspension and capsules, (**A**) dissolution in buffer at pH 1.2; (**B**) at pH 4.5 and (**C**) at pH 6.8.

We then questioned whether the TMZ suspension and the TMZ capsules are bioequivalent or whether dose adjustments are required. An in vitro dissolution assay was performed using the suspension and Temodal^®^ capsules in order to support the equivalence between the two formulations. According to the regulatory guidelines for in vitro demonstration of bioequivalence for BCS-class 1 drugs [12], two formulations dissolving at least 85% of the drug substance within 30 min are deemed bioequivalent. As shown in Figure 3, total dissolution of TMZ was achieved in the 3 pH media (pH 1.2, pH 4.5 and pH 6.8) within 5 min for the suspension and 15 min for the TMZ capsules, the delay between suspension and capsules is related to the dissolution time of the capsule. These data support the bio-equivalence between TMZ suspension and TMZ capsules.

### 2.7. TMZ Suspension’s Palatability Optimization

During this period, 70 patients were treated with the hospital-compounded suspension of TMZ prepared with or without various flavouring agents. The median age of the population was 5.0 years, ranging from 1 to 58 years. As expected, most participants were young patients facing difficulties with the administration of TMZ capsules (38 out of 70 were below 6 years for which this solid form is not adapted [1], whereas four patients were adults (19, 40, 54 and 58 years) facing swallowing difficulties related to their oncologic condition. Thirty-seven patients were male and 33 were females.

All patients treated with the TMZ suspension were offered to participate. Among the 70 patients, 12 had a nasogastric tube and therefore could not participate in the evaluation of the palatability of the suspension. Eighteen patients refused to participate in the evaluation of the palatability. Two patients received and evaluated two different flavours. One patient received two flavours (mint and cola) but completed the evaluation for the cola flavour only. In total, 40 patients participated, and 42 evaluations were completed using a 20-point scale combining auto-evaluation and hetero-evaluations (see Materials and Methods).

Three patients were treated with the suspension without any flavouring and this was not taken. Subsequently, three children aged 6 to 9 years were given a suspension prepared with SyrSpend cherry but again the taste was not very good with a score of 7.3/20 on our scale. Given the strong rejection of this formulation, only three patients completed a treatment course with the cherry-flavoured suspension and it was decided to test additional taste-masking agents.

Thirty-three patients were then treated with the mint-flavoured preparation, including four adults. Seventeen patients completed the evaluation (paediatric patients aged 3–11, two adults aged 40 and 58 years). The overall score was 11.9/20 (12.3/20 when excluding adult patients), ranging from 7/20 to 17/20. Three patients (aged 9, 9 and 19) out of all patients treated stopped the use of the suspension because of the taste and tried to use the capsules. After some discussions with the nurses and the caregivers, it seemed that the palatability of the mint-flavoured suspension could be improved with another flavouring agent (some children did not appreciate the mint flavour and found the suspension had a bitter taste). Therefore, lime and cola were selected afterwards according to the European Paediatric Formulation Initiative (EuPFI) recommendations [13] to continue the study.

Eleven patients (aged 2 to 14 years) were then treated with the lime-flavoured suspension and completed the evaluation. The overall score was similar to the mint-flavoured suspension (12.2/20). This score was considered not satisfactory.

**Figure 4:**
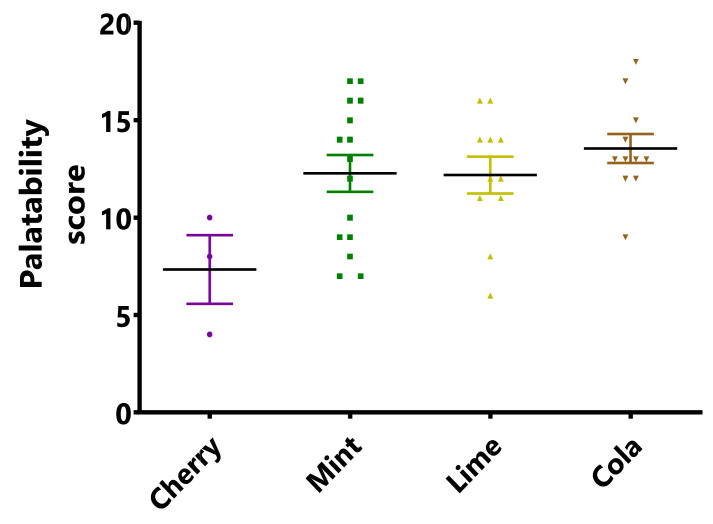
Palatability scores of cherry, mint, lime and cola flavoured TMZ suspensions. Cherry scored was significantly inferior to the other three (*p* < 0.05), there was no significant difference between the other 3 groups.

Eleven patients (aged 2 to 14 years) were then treated with the cola-flavoured suspension. The cola-flavoured preparation had the best mean acceptability score of 13.5/20 ranging from 9 to 18. Only a 4-year-old patient had a score below 10. This flavour had the highest mean score (13.5/20), the highest maximum score (18/20) and the highest minimum score (9/20). In total, 91% (10/11) “accepted or appreciated” the cola-flavoured suspension according to our evaluation scale. No patient stopped the treatment as a result of the taste of the suspension. It was concluded that this flavouring agent was able to efficiently mask the taste of TMZ and that the cola-flavoured suspension was well accepted by the paediatric population (Figure 4).

### 2.8. Real-Life Experience 2015–2020

**Figure 5:**
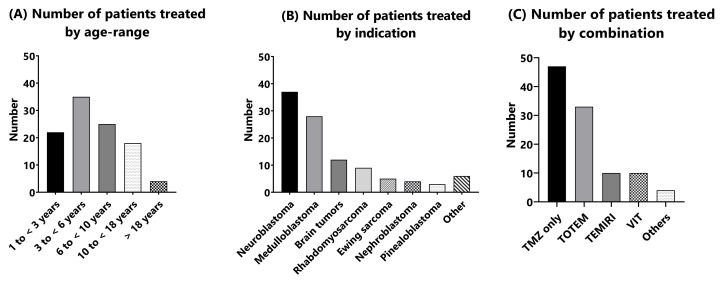
Number of patients treated with TMZ suspension by age range (**A**), per indication (**B**) and per combination (**C**) (TMZ only: children treated with temozolomide suspension in monotherapy; TOTEM: association of temozolomide and topotecan; TEMIRI: association of temozolomide and irinotecan; VIT association of vincristine, irinotecan and temozolomide; Others: associations corresponding to temozolomide associated with other drugs).

From April 2015 to December 2020, 104 patients (58 boys and 46 girls) facing difficulties with the ingestion of TMZ capsules were treated with the TMZ suspension at Gustave Roussy cancer centre without change of the medical practice. The median age at the start of treatment with TMZ was 5.3 years, with 55% (*n* = 57) below 6 years (Figure 5A). The most frequent malignancy was neuroblastoma (*n* = 37, 36%), which reflects the key role of TMZ in the treatment of these patients (Figure 5B). The median age of patients with neuroblastoma was 4 years, with 26 (70%) patients below 6 years of age (for whom the use of capsules is not adapted), among whom 13 were below 3 years. The second most frequent cancer indication was medulloblastoma (*n* = 28, 27%) with a median age of 6.1 years (14 children below 6). Other indications were brain tumors (*n* = 12, 11.5%, median age 8.3 years), rhabdomyosarcoma (*n* = 9, 8.7%, median age 4.8 years) and Ewing sarcoma (*n* = 5, 4.8%, median age = 11 years) and other indications (*n* = 13).

Forty-seven patients received TMZ monotherapy (STUPP, maintenance in PNETHR + 5 or palliative care), 33 received TMZ and topotecan (TOTEM; among them, 10 received a few subsequent cycles of TMZ monotherapy), 10 received TMZ and irinotecan (TEMIRI) and 10 received vincristine-irinotecan-TMZ (VIT) protocol (Figure 5C). In total, 602 cycles were delivered, including 466 with the suspension (some patients started their treatment with TMZ capsules and switched to the suspension after few cycles). The mean number of TMZ cycles (capsules and suspension) received by patients was 5.8 (median = 4, range 1–32), whereas the mean number of cycles received with the suspension was 4.5 (median = 3, range 1–24).

Typical target dose volumes for paediatric liquid [14] formulations are < 5 mL for children under 5 years and < 10 mL for children of 5 years and older. In our cohort (104 patients, including 4 adults), 50 paediatric patients were below 5 years with a median initial TMZ dose of 80 mg corresponding to 4 mL of TMZ suspension. A further 50 paediatric patients were 5 years and older with a median TMZ dose of 120 mg corresponding to 6 mL of TMZ suspension. For most patients, the administration of the 20 mg/mL TMZ suspension met the EMA target dose volume recommendations. Only 2 of the 104 paediatric patients, aged 17.4 and 17.9 years at the initiation of TMZ treatment, received a volume > 10 mL (i.e., 12.5 mL for both).

This work was not aimed at evaluating efficacy or safety. However, we describe the tolerance of TMZ in the treated population by assessing the number of cycles delayed, among which the number of cycles delayed for toxicity (collected for all patients but one who received 12 cycles of TOTEM). Among the 590 cycles assessed for tolerance, only 100 (16.9%) were delayed, among which only 67 (11.4%) were delayed for toxicity. This data is not different from previous studies [15,16] and confirms that TMZ is well tolerated with manageable side effects. As expected, the proportion of cycles delayed as monotherapy was lower when compared to combination treatments (11.7% vs. 21.8%, respectively).

Although it was not aimed at comparing the safety of the two different forms (suspension versus capsules), there was no unexpected safety signal with the use of the suspension according to the clinicians involved in the care of patients. Only one patient had a potential formulation-specific safety event (a grade 2 mucositis), which led to the recommendation of rinsing the mouth with a glass of water after administration of the suspension. No other case of mucositis was subsequently reported.

## 3. Discussion

TMZ is an important method of chemotherapy for the treatment of certain pediatric oncology conditions. It has become standard chemotherapy for the management of relapsed or refractory neuroblastoma in which it is often combined with other molecules such as irinotecan, topotecan or anti-GD2 immunotherapy. It is also part of the treatment armamentarium for relapsed medulloblastoma and recurring gliomas. Despite being an important molecule for the treatment of cancers affecting very young patients, there is currently no formulation adapted to children. Consequently, caregivers are instructed to open TMZ capsules and dispense their content in drinks or soft food, which is clearly not acceptable as it results in exposure to a cytotoxic drug, unprecise delivery and dosing, as well as uncontrolled stability of the active molecule.

To address this issue, we developed a hospital compounded suspension of TMZ. According to the EMA, liquid formulations are most appropriate for younger pediatric patients who are unable to swallow capsules or tablets and the dose volume is a major consideration for the acceptability of a liquid formulation [1]. The choice of making a suspension rather than a suspension made it possible to produce a liquid form, which limited the degradation of temozolomide by hydrolysis. Moreover, a suspension allowed reaching a high TMZ concentration that facilitated the administration of small volumes in line with the EMA recommendations. Furthermore, we were able to achieve 8-week-stability under refrigerated conditions (and 10 days in ready-to-use oral syringes), which is compatible with a home-based treatment. One of the problems in the development of oral forms and especially in syrups, solutions or suspensions is to think carefully about compliance. This is especially important in oncology where failure to take the treatment leads to a high risk of progression or relapse of the disease. In the case of cytotoxics, the metallic taste is expected and difficult to mask, that is why it was important to monitor these parameters. We performed a palatability study aiming at identifying the best taste-masking agent using a new composite scale. We identified a flavouring agent (cola) with an excellent ability to mask the taste of the TMZ suspension, which otherwise systematically leads to poor acceptability or refusal of the treatment.

Over a 5–year period in a single cancer centre (Gustave Roussy), 466 cycles were delivered with the suspension, further highlighting the critical need for a pediatric formulation of TMZ. The patients received a median number of 5.8 TMZ cycles. Some patients benefited from the therapy for a very long period and remained under TMZ treatment for up to 32 cycles. Although safety was not evaluated using clinical standards, there was no unexpected safety signal with the suspension, suggesting that TMZ suspension has a similar safety profile compared to capsules.

In conclusion, we report here the successful development of an oral liquid form of TMZ, which fills an important medical need for young patients with cancer. We developed a composition that allows high TMZ concentration, efficiently masks the TMZ taste and exhibits stability compatible with treatment in an outpatient setting. The successful development of the hospital compounded suspension led to the development of an industrialized formulation currently under clinical evaluation.

## 4. Materials and Methods

### 4.1. TMZ, Excipients and Formulation

The TMZ oral suspension was prepared from the capsule content (Temozolomide, SUN Pharmaceutical, India or MSD, NJ, USA), a Syrup-suspending vehicle, SyrSpend^®^ (Fagron, Belgium), Povidone K30 (Fagron, Belgium), and Citric Acid (Fagron, Belgium) Sucralose (Merck, Germany) Cola, Mint, Lime flavours (International Flavors and Fragrances, New-York, NY, USA). Syrspend^®^ is a ready-to-use agent comprising a suspending agent which contains modified food starch, simethicone as an antifoaming agent, sucralose as a sweetening agent and a pH adjusting agent and water.

The TMZ suspension was prepared according to the following instructions. TMZ 250 mg capsules were opened and mixed with povidone K30 in a mortar for 2 min. Citric acid was then added, and the mixedure was triturated for 5 min. Then, the mixed powder is transferred into a beaker then SyrSpend^®^ was gradually incorporated under constant agitation for 15 min at 500 rpm with bench mixer Turbotest^®^ (VMI Saint Hilaire de Loulay, France). Sucralose and aroma were diluted in sterile water then added and then mixed again for 3 min.

The preparation is carried out in a vacuum room classified as ISO 7, and under an isolator, which is itself under vacuum to guarantee the protection of the staff.

### 4.2. Reagents and Reference Standards

Acetonitrile (ACN) and water were purchased from Fisher Scientific^®^. All solvents used were HPLC grade. TMZ and AIC, the major degradation product, were supplied by Sigma-Aldrich^®^ (Saint-Quentin-Fallavier, France).

### 4.3. Pre Formulation Study

A pre-formulation compatibility study of triturated TMZ and dry excipients mixedure was performed using differential scanning calorimetry (DSC) analysis. The DSC test was performed on a Q1000 TA Instrument (Guyancourt, France) in hermetic aluminium pans. The temperature range investigated was between 20 °C and 230 °C. The heating rate chosen was 10°C/min. The experiments were carried out in nitrogen enthalpies of fusion and melting points were measured by carrying out five different analyses. The experiment was performed 6 times. TMZ 250 mg capsules (ACCORD healthcare^®^ (Lille, France) and TMZ powder (Excella GmbH, Nuremberg, Germany) were used as comparators.

### 4.4. RP-HPLC Method

The analytical method used was reversed-phase high-performance liquid chromatography (RP-HPLC, 1260 Infinity, Agilent^®^) with isocratic conditions. The analytical column was a reversed-phase C18 (250 mm × 4.6 mm, 5 μm, Waters). The mobile phase was composed of 90% of orthophosphoric acid 2.4 buffer, and, 10% of acetonitrile, adapted from Jedynak et al. [17]. Detection was performed through an ultraviolet detection array diode spectrometer at 254 nm. The autosampler was set at 4 °C and the column was maintained at 20 °C.

The method was validated for dosage in a SyrSpend^®^ matrix in accordance with the ICHQ2. TMZ and AIC calibration samples were diluted in 0.5% acetic acid water, respectively, from 0 to 200 µg/mL and 0 to 2 µg/mL. Samples and blanks were prepared under the same conditions. Each tested sample was centrifuged for 10 min at 3500 rpm and diluted at 1/200 in acid water (0.5% of acetic acid) before analysis. Experiments assessing TMZ suspension stability were performed with both brands of TMZ (Sun Pharmaceuticals and MSD) and were replicated 9 times.

### 4.5. Forced Degradation

Experiments were performed in accordance with ICH guidelines Q1 A (hydrolytic conditions, oxidative conditions, thermal stress conditions and photolytic conditions):

1. Hydrolytic conditions: the TMZ was incubated with HCl (1 N) or NaOH (1 N) for 30 min at room temperature;
2. Oxidative conditions: the TMZ was incubated with 1% H_2_O_2_ for 30 min at room temperature;
3. Thermal stress conditions: incubation for 1 day at 60 ± 2 °C without light exposure;
4. Photolytic conditions: Samples were artificially aged in a Q-SUN XE−1 xenon arc chamber (Labomat, Saint-Denis France) that reproduces full-spectrum sunlight. The conditions were 96 h of irradiation with a power of 6.8 mW/cm^2^ from 300 to 400 nm or 127 klx. The test was performed at 25 °C for 2, 4 and 24 h which reproduced 1, 2 and 16 months of light exposure.

### 4.6. pH and Osmolality Analyses

Osmolality (Osmometer Camlab, Cambridge, United Kingdom,) and pH (pH meter Methrom, Zofingen, Switzerland) were measured in triplicate every 3 days over 60 days.

### 4.7. Compatibility with Oral Syringes

TMZ suspension is intended to be administered using oral syringes for 5 days every 21 or 28 days according to current protocols. Hence, the treatment can be delivered as a pharmacy prepared syringes for each day or as a suspension bottle. In all cases, a minimum of 5-day stability in the syringe is required. The compatibility and stability of the TMZ suspension in oral polypropylene syringes were evaluated over a 10-day period.

The compatibility of the TMZ suspension and the polypropylene syringes was tested in two steps. Polymer degradation products and additive migration were analysed by Soxhlet forced extraction with dichloromethane. The leachables profile of polypropylene was compared before and after contact with TMZ suspension for up to 10 days at room temperature and 2–8 °C. Then, migration of polypropylene leachables was explored in the TMZ suspension after contact. All extracted solutions were analysed by RP-HPLC paired with a diode array detector with a 100% ACN mobile phase. This chromatographic method allows full detection of oligomers, additives and low molecular weight compounds. In parallel, after 10 days, 6 syringes were emptied then rinsed, then the TMZ residues were extracted and the residues were analysed by RP-HPLC.

### 4.8. Administration through Nasogastric Tube

The feasibility of administering the TMZ preparation via a nasogastric feeding tube was evaluated in simulated conditions. A nasogastric feeding tube was used for the experiment (Ansell^®^, Albi, France) Corflo 8 CH polyurethane nasogastric tube coated with a hydrophilic lubricant. Nasogastric tubes (*n* = 12) were lubricated as recommended by the manufacturer, and then 10 mL of TMZ suspension (200 mg) were administered. The residual concentrations of TMZ were measured after 1 or 2 rinses with 5 or 10 mL of water. After the first or second rinse, an elution of the probes took place to remove all temozolomide potentially remaining in the tubes.

### 4.9. Dissolution Test

A dissolution test was performed comparing TMZ capsules and TMZ suspension according to the 10th Edition of the European Pharmacopeia. The test was performed with a Modular Dissolution System (Sotax paddle) at 37 °C filled with distilled water and hydrochloric acid solution to reach pH, 1.2, acetate buffer for pH 4.5 and phosphate buffer for pH 6.8, the total volume was 900 mL and the agitation speed was 100 rpm. One hundred milligrams was used for the dissolution test (i.e., 1 cap of 100 mg is equivalent to 5 ml of the suspension). Samples were collected at 0, 2, 5, 10 and 20 min and analysed by RP-HPLC in order to determinate the kinetic dissolution curves of both pharmaceutical forms (*n* = 6).

### 4.10. Microbiological Study Analysis

The microbiological study (*n* = 6) was performed in conformity with the European pharmacopeia 5.1.4. microbiological quality of non-sterile pharmaceutical preparations and substances for pharmaceutical use. The microbiological examination of non-sterile products was undertaken including the total viable aerobic count. The microorganisms tested (Staphylococcus aureus CIP 4.83, Bacillus subtilis CIP52.62, Pseudomonas aeruginosa CIP 82118 and *Candida albicans* CIP 48.72) were obtained from the Biological Resource Centre of Institut Pasteur (France) and the growing medium (Trypticase Soy Agar-TSA and Malt Chloramphenicol Agar-MCA, respectively, incubated at 37 °C and 30 °C) was purchased from Biomérieux^®^ (Craponne, France).

Positive control solutions with or without TMZ were artificially inoculated (with the above-mentioned microorganisms) at day 0 and analysed after 2 and 7 days, to prove that TMZ does not inhibit microbiological growth. The tested samples were diluted 1:10 in sterile water, and then soaked in different media and analysed at D0, D1, D7, D14, D28 and D60. According to the 10th Edition of the European Pharmacopoeia, acceptance criteria for the microbiological quality of non-sterile dosage forms are:

1. A total aerobic microbial count (TAMC) below 10^2^ CFU/mL;
2. A total combined yeast/mould count (TYMC) below 10^1^ CFU/mL;
3. Absence of Escherichia coli.

### 4.11. Statistical Analysis

All experiments were replicated at least 6 times. The results are expressed as means +/− standard deviation. Data were analysed by Prism 8.0.2^®^ software using the Student *t*-test and Anova regression tests.

### 4.12. Palatability Study

During the development of oral solutions or suspensions, one of the most important challenges is to ensure its good observance. A key point of treatment observance is the palatability of the drug product, which is even more critical with pediatric patients who are very reluctant to swallow badly tasting products. Poor palatability can lead to poor compliance. Usually, the global palatability of a drug product is only evaluated by a hedonic scale of facial expression [18,19,20,21]. Children are considered able to participate in taste assessment from 4 years old [22].In addition, their ability to understand and follow instructions is sometimes limited. They may also lose interest in the test or have difficulty concentrating throughout the duration of the test. Their test failure rate can be up to 50% depending on the design and duration of the test. It is also common for them to be unable to communicate their feelings and preferences. Therefore, a suitable evaluation scale for palatability of pediatrics pharmaceutical formulations is required to develop these products appropriately. Given the difficulty of evaluating young children with neurologic disease, we developed a new composite scale based on published evaluation scales (24).

This new evaluation scale was used to evaluate the palatability and acceptability of the TMZ suspension.

**Figure A1:**
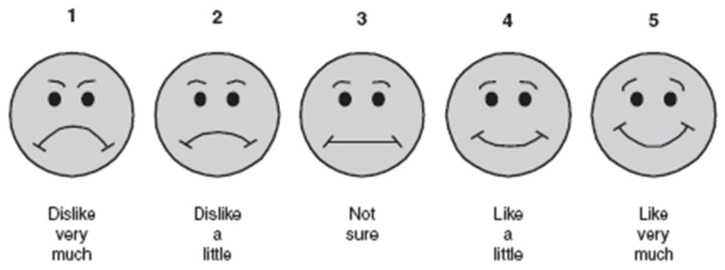
Hedonic Five Scake Facial Expression.

The palatability study aimed at evaluating the palatability and acceptability of the TMZ hospital-compounded suspension prepared with or without one of four different flavours. The initial choices of flavours were oriented toward cherry and mint for their known ability to mask metallic tastes. Lemon and cola were tested a second time. All flavours were selected after discussion with paediatricians in accordance with EuPFI recommendations. The study was conducted by the paediatric diseases ward of Gustave Roussy Cancer Campus on a prospective non interventional cohort with the agreement of the Ethics Committee No.20220408. A minimum of 10 patients per group was planned for the palatability evaluation. The evaluation of the palatability was realized at the end of the first treatment course or during the second treatment course of TMZ. All children treated with the TMZ suspension were offered to participate to the study. Patients who completed at least one course of treatment were asked to complete the palatability evaluation form on the first day of the first or second cycle of treatment. Patients with nasogastric tube were excluded from the palatability evaluation. A 20-point scale was created by combining scores of auto-evaluation scales and hetero-evaluation scales already published. The score is the addition of four distinct evaluations of the palatability:

Patients themselves performed two auto evaluations:

- Rating on a hedonic scale (5 points) (Figure A1);
- Spontaneous verbal response (5 points) Table A1);
- The parents or the caregivers performed two hetero evaluations;
- Rating on a hedonic scale (5 points), the same as used by the child (Figure A1);
- Patients’ reaction monitored by a third party who could be either a pharmacist resident or a nurse (5 points) (Table A2).

The 20-point score evaluation was tested using 2 placebo suspensions (all excipients without TMZ and with or without cherry flavour) on thirty volunteers aged 2 to 13 years old treated at Gustave Roussy cancer centre. All data could be properly collected for all placebo-treated patients and the scores were used to define the score’s interpretation. Interviews of patients confirmed that all children with score below 5 would refuse treatment and all patients with score of 15 or above would accept treatment.

## 5. Conclusions

We have developed a reliable solution, making it possible to best treat patients requiring temozolomide with the means available in the hospital. the preparation produced has shown that it was well tolerated and well accepted, which is a real challenge for oral anticancer drugs.

## Figures and Tables

**Table 1:** TMZ suspension composition. Quantity of TMZ and excipients per 100 mL (1 bottle) or per mL of suspension.

	Quantity (mg or mL)/100 mL of Suspension	Quantity/mL of Suspension
TMZ 250 mg capsules	2000 mg	20 mg/mL
Lactose (present in TMZ caps 154.3 mg)	1234.4 mg	12.34 mg/mL
Povidone K30	500 mg	5 mg/mL
Citric acid (anhydrous)	150 mg	1.5 mg/mL
Purified water	3 mL	30 µL
SyrSpend^®^	q.s 100 mL	q.s 1 mL
Flavour	400 mg	4 mg/mL
Sucralose	50 mg	0.5 mg/mL

**Table 2:** TMZ and AIC assay method and validation results.

	RT	λ	Regression Curve	R^2^	Linearity	Regression	LOD	LOQ	Recovery
TMZ	4.38 min	254 nm	Y = 39,654 x − 3874.95	0.9991	0.2–100 µg/mL	*p* < 0.001	0.17 µg/mL	0.20 µg/mL	99%
AIC	1.80 min	254 nm	Y = 83,965.2 x + 1227.82	0.99	0.13–2.0 µg/mL	*p* < 0.001	0.066 µg/mL	0.13 µg/mL	97%

**Table 3:** Residual TMZ extracted from nasogastric tubes after administration of 200 mg TMZ. After the first or second rinse with 5 or 10 mL of water (following nasogastric tube supplier recommendation), TMZ was extracted from nasogastric tube and was measured with HPLC, the detection limit was 17µg.

Washing	10 mL Rinsed Group		5 mL Rinsed Group	
	1st Rinsing	2nd Rinsing	1st Rinsing	2nd Rinsing
Residual TMZ	20 µg (0.01%)	<LOD	75 µg (0.0375%)	10 µg (0.005%)

## Data Availability

Data is contained within article and Supplementary Materials.

## Supplementary Materials

The following are available online at https://www.mdpi.com/article/10.3390/ph15050555/s1, Figure S1: Differential Scanning Calorimetry Curves of TMZ.

## Appendix A

**Table A1:** Spontaneous Verbal Response adapted from Sjovall et al. [19].

1	2	3	4	5
It’s disgusting	It’s not good	No comments	Great	Amazing
Yuck	I do not like it	I do not know	Yummy	Super good
Argh	Too sweet	No answer	It’s good	Very good
Brrr	I do not like the taste	Silence	Mmmmh	Awesome
Aawfull	Too strong	Not too bad	I Like it	Very sweet
Horrible	There is an after taste	Medium	Can I take again?	Like a candy
		It’s ok	I love it	
		OK		
		Ordinary		
		Not particularly good		
		A bit strong		

**Table A2:** Hetero-Evaluation by a Third Party.

1	2	3	4	5
Vomit	Strong grimace	Swallows hard	Indifference	Smiles
Spits out	Pulls the tongue	Forced smile	Does nothing	Nodding
Does not swallow	Disgust sign	Does not head	Shoulder shake	Wanting more
Shouting	Inflates the cheeks	Slight grimace		
Cry	Hits			
