# Supplementary material for: Development of a Hospital Compounded, Taste-Masked, Temozolomide Oral Suspension and 5-Year Real-Life Experience in Treating Paediatric Patients
Source: Pharmaceuticals (Basel). 2022 Apr 29;15(5):555. doi: 10.3390/ph15050555 (PMC9146721; doi:10.3390/ph15050555)
Supplement: Supplementary file 1 [file pharmaceuticals-15-00555-s001.zip › pharmaceuticals-1667040-supplementary.pdf]

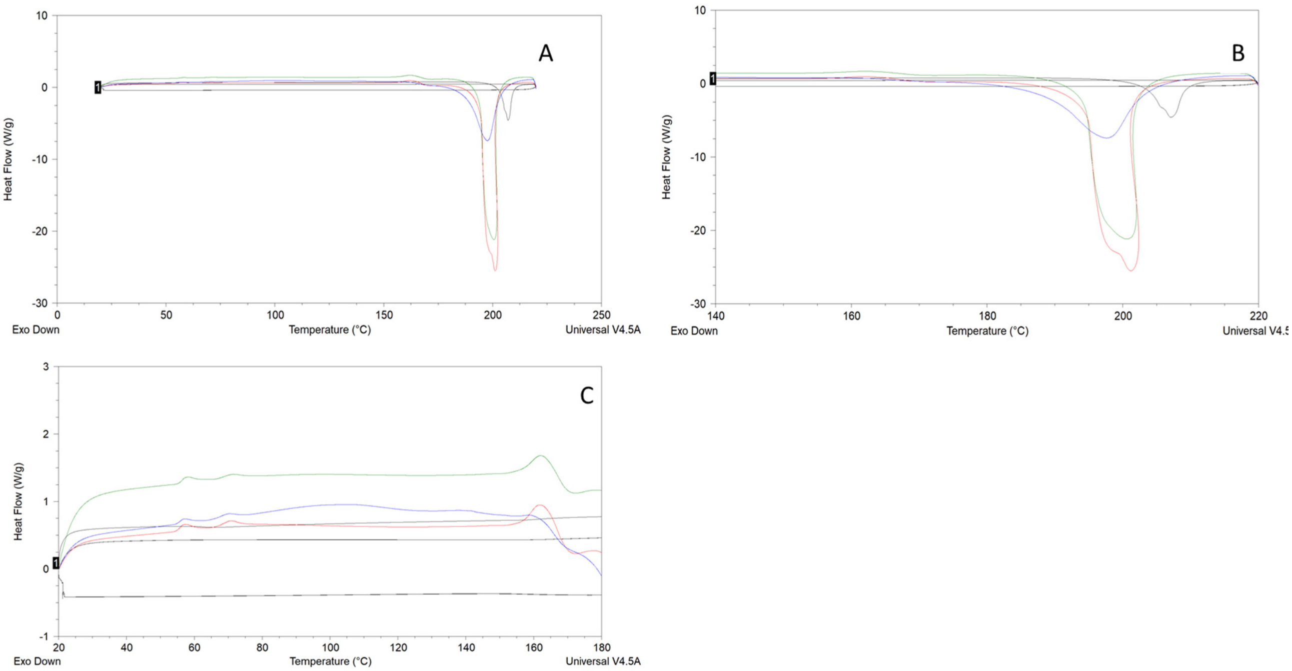

Figure S1. Differential Scanning Calorimetry Curves of TMZ EXcella® in black, Sun's TMZ caps in green, Accord Healthcare caps in red and our mixed in powder in bleu. (**A**) globl DSC curve shows Recrystallisation peak of TMZ around 200 °C present for forms with excipients. The presence of excipients does not allow the visibility of the TMZ peak (212 °C). However, there is no difference between the marketed capsule forms and the hospital preparation form. (**B**) A zoom for the recrystallisation peak. (**C**) A zoom on the beginning of the DSC curves showing three melting peaks are visible near 57 °C, 72 °C and 162 °C, which are absent from the raw material. These peaks correspond to the presence of excipients associated with the dosage form. There is no significant difference between the plots of the SUN, Accord or hospital mix capsules.
